# GutLogo: Agent-based modeling framework to investigate spatial and temporal dynamics in the gut microbiome

**DOI:** 10.1371/journal.pone.0207072

**Published:** 2018-11-09

**Authors:** Charlie Lin, Joshua Culver, Bronson Weston, Evan Underhill, Jonathan Gorky, Prasad Dhurjati

**Affiliations:** Chemical and Biomolecular Engineering, University of Delaware, Newark, DE, United States of America; Helmholtz Centre for Infection Research (HZI), GERMANY

## Abstract

Knowledge of the spatial and temporal dynamics of the gut microbiome is essential to understanding the state of human health, as over a hundred diseases have been correlated with changes in microbial populations. Unfortunately, due to the complexity of the microbiome and the limitations of *in vivo* and *in vitro* experiments, studying spatial and temporal dynamics of gut bacteria in a biological setting is extremely challenging. Thus, *in silico* experiments present an excellent alternative for studying such systems. In consideration of these issues, we have developed a user-friendly agent-based model, GutLogo, that captures the spatial and temporal development of four representative bacterial genera populations in the ileum. We demonstrate the utility of this model by simulating population responses to perturbations in flow rate, nutrition, and probiotics. While our model predicts distinct changes in population levels due to these perturbations, most of the simulations suggest that the gut populations will return to their original steady states once the disturbance is removed. We hope that, in the future, the GutLogo model is utilized and customized by interested parties, as GutLogo can serve as a basic modeling framework for simulating a variety of physiological scenarios and can be extended to capture additional complexities of interest.

## 1 Introduction

The symbiotic relationship between the gut microbiota and its human host plays an integral role in metabolic function, energy extraction/storage, and the proper development of the host immune system [1]. It has become apparent in recent years that an imbalance in this relationship due to the effects of heredity, diet, hygiene or other environmental factors can have far-reaching implications for human health [1]. Several publications have identified possible healthy and diseased states of the human gut microbiome, prompting an increased interest in the gut microflora as a diagnostic tool [2]. A significant variation in gut microflora has been found in patients with afflictions as diverse as autism, Celiac’s disease, type II diabetes, obesity and irritable bowel syndrome [1]. For example, papers by Finegold et al. have suggested a correlation between the presence of late-onset autism and an imbalance of *Clostridium* and *Desulfovibrio* populations [3, 4]. Further investigation into these observations via clinical studies is limited by the lack of convenient sampling methods and the complexity of the gut microbiota. Sampling gut-bacteria populations consists of either a representative sampling from fecal samples or an invasive procedure to extract tissue [5]. Since both of these methods are only capable of a small number of static, discrete measurements, neither is optimal for examining the spatial and temporal complexity of the gut microbiota. These spatial and temporal insights are important for a comprehensive understanding of the human-microbiome interaction, since the microbiome fluctuates based on transient environmental factors and since a bacterial imbalance at one position in the gut could precipitate a local inflammatory response. Additionally, sequencing of samples and data analysis is a limiting factor due to the added burden on funding, time and resources. As a further impediment to clinical trials, the Human Microbiome Project observed considerable variability in the gut-bacteria population profiles of apparently healthy individuals [6]. Since unrelated individuals may share less than 30% of strains in their microbiota, clinical trials must sample a large population to obtain a representative profile of healthy and diseased gut states [7].

The challenges of data collection, combined with the requirement of a large sampling population, incentivizes the creation of a generalizable gut-microbiota model. A well designed and validated model could be used to study the dynamics of many hypothetical situations in a fraction of the time and cost needed to do a clinical study. For example, researchers would be able to test the effects of an unhealthy diet or pathogen on the bacterial community in “real time”. Importantly, such a model would allow for the ethical testing of many what-if scenarios that could not be investigated in human trials. One large challenge in developing a reliable model is validation, as large heterogeneity of microbiota within human and mice populations [6, 8] makes determining shifts in populations quite challenging. Studies where germ-free mice are infected with specific bacteria offers a good place to start, but experiments will likely need to be designed with the intention of validating models. The biological outcome of the model can be validated more readily than its mechanistic assumptions, since mechanistic validation required *in vivo* data whereas a physiological response to a given disturbance is easily documented. For now, preliminary models of human gut dynamics can still be useful in hypothesis generation for future medical studies.

While some systems are highly conducive to differential equation (DE) modeling, an accurate DE model of the gut microbiota would require considerably more fundamental knowledge about the system interactions than is currently available. For such complex systems in which the interactions between “agents” (in this case bacteria) have not been formalized into mathematical equations, agent-based models prove to be particularly useful [9]. Such a framework allows researchers to encode the intricacies of a multi-bacterial system into a series of relatively simple rules that can be tuned as new and more accurate information arises. The number of bacterial species in the system can also be increased to more closely match the physiological reality of the microbiome without necessitating a reworking of the previous interaction statements. If adding species in a DE model, the mathematics quickly becomes prohibitively complex as interactions between the newly incorporated bacteria and each other species in the model must be formalized mathematically. This is as opposed to an ABM, in which knowledge of a small number of species-specific parameters such as metabolite production, growth rate and metabolite consumption allows for the incorporation of new species via a series of relatively simple if-then statements. This ease of tuning and modification makes an ABM both a flexible and generalizable modeling framework.

In consideration of the difficulties facing microbiota research and the utility of agent based modeling, we have developed GutLogo, a modeling framework of microbes residing in the intestinal tract. The two-dimensional surface of the model represents the inner wall of the ileum, and the current framework includes four significant microbial genera competing for various carbohydrates and interacting with both the flowing fluid of the gut and the intestinal wall. Through this model, users can alter parameters such as initial bacterial populations, nutrient intake, flow rate and metabolite production to observe the change’s effect on bacterial population dynamics. Such simulations can yield informative results that may help direct hypothesis formation for researchers in the medical field. Some initial observations, described in this paper, suggest that *Bifidobacterium* probiotics only alter the gut microbiota during their period of use, and that variations in glucose intake may dramatically alter bacteria levels. In addition, the model predicts that a decreased intestinal flow rate associated with constipation has only transient effects on the microbiota, while diarrhea has prolonged implications for the population profile. By utilizing and building off of the GutLogo framework, researchers can use such simulations to gain insight into gut bacteria dynamics.

## 2 Related work

There are several existing bacterial simulation platforms. They come in two types: intracellular models and population-level models.

Intracellular models commonly use molecular-level interactions to simulate the inner mechanisms of one cell. An example of an intracellular simulator is Smoldyn [10], which models molecular collisions, diffusion, and reactions to obtain spatial and stochastic detail of cellular mechanisms. While it is possible in theory to extend intracellular simulations to model bacterial populations, such an endeavor would be too computationally intensive to provide results in a reasonable amount of time. Therefore, simplifying assumptions are necessary for the creation of a population-level simulator.

Many population-level simulators, which model whole-population dynamics of cell communities, have been released that model intercellular and, sometimes, intracellular interactions with great depth. One such model, AgentCell [11], wrapped output from Stochsim [12], an intracellular simulator, into a multi-cell simulation. This simulation, however, is computationally taxing since each cell requires an independent core. This severely limits the number of cells that can be modeled. More recent models and frameworks, such as BSim [13] and BNSim [14], have increased the possible spatial complexity and number of bacteria capable of being modeled. Still, the scale of these simulations is only on the order of millimeters because of their focus on the accuracy of micro-scale effects. These models also require the user to have a programming background to use in research since the simulators lack simple graphical interfaces and must be tuned and altered to simulate the gut. Shashkova et al. recently released a simulator that specifically models two bacterial species in the human gut microbiome and the effect of antibiotics [15]. Their implementation shows the feasibility of a macro-scale model for the gut microbiome. The model goes into great depth, incorporating factors such as toxins, feedback loops, and mutation while maintaining reasonable hypotheses. However, the complexity of the model forced a loss of generality and ease of use. There are a number of cell-based modeling platforms, such as Morpheus and Chaste, that incorporate spatial discretization into a general multi-scale model of specific biological environments [16, 17]. These are very powerful tools for complex biological systems, however both utilize purely mathematical and deterministic approaches that are not conducive to modeling the highly variable gut microbiome.

To have a simulator that can provide insight into gut microbiome dynamics while maintaining high levels of accessibility, a general framework with an adequate user interface is required. Therefore, we use the NetLogo [18] agent-based framework for our model, as it has a simple graphical interface and easy-to-learn programming language. These attributes will facilitate the program’s widespread use in medical research, allowing users to modify initial conditions and gut behavior for the purpose of investigating “what-if” physiological scenarios.

## 3 GutLogo overview

GutLogo is an agent-based model created in NetLogo, designed to model variations in gut operating parameters and simulate their subsequent effects on the microflora population dynamics. It employs a bottom-up modeling approach, basing agent behavior on biological principles rather than modeling empirical data of gut microbiome dynamics. The agent-based modeling approach, like bacterial co-populations in general, is stochastic in nature, since each action is decided by a set of probabilistic rules that results in unique model dynamics for each simulation run. GutLogo was refactored from the Weston et al. model; a model motivated by the theory that imbalances between the populations of *Desulfovibrio*, *Bifidobacterium* and *Clostridium* in the gut may contribute to the development of autism spectrum disorders [19, 20]. The model was developed to study the dynamics of these three populations by considering the direct interactions between these bacteria and their metabolic relationships. Weston et al. used the model to evaluate how these populations might shift due to different biological factors, such as lysozyme concentrations and differing nutrient levels, and evaluated these results in the context of autism [19]. The original model proved both the feasibility of creating a useful simulation of gut bacteria dynamics and the flexibility of the agent-based method. We specifically chose to continue development on this agent-based model due to its ease of use and ability to simulate from limited data. The GutLogo model diverges from the Weston et al. model in several ways. Firstly, while the focus of the Weston et. al. model lies on capturing the dynamics of populations related to autism, the focus of GutLogo is to demonstrate the utility and ease of agent-based models in the context of studying microbial populations in the intestinal tract. Thus, we neglect direct interactions between bacteria, and set bacteria replication rates to constant values (as opposed to the variable replication rate of the Weston et al. model). This increases the stability of the model and allows us to focus more on the other aspects of the system. Most importantly, we expand on the model by creating a map with the appropriate dimensions of the ileum, taking into account the unidirectional flow in the intestine and by scaling time-dependent factors such as flow rate and transit time to realistic values to increase the model’s relevancy. This is a significant improvement over the Weston et. al. model, which did not focus on spatial features of the gut, and instead modeled bacteria as freely diffusing agents and had periodic boundaries on both the x and y axis. Finally, we add a fourth bacteria genus, *Bacteroides*. Although this genus acts identically to *Clostridium* in our simulations, it serves as a great benchmark for the impacts of initial conditions on final populations as these genera are simulated with different initial conditions.

### 3.1 Biology

The gut microbiome contains hundreds to thousands of different bacterial species that interact with each other through competitive, symbiotic, parasitic, and commensal interactions. In spite of the large number of studies examining the shifts in relative abundance and metagenomic functions of these bacterial communities, there is limited work to date examining the interactions of multiple bacterial families or species in any mechanistic fashion. Therefore, all interactions in this agent-based model must be inferred from literature about each bacterial subtype in isolation. While the interactions of the four bacterial subtypes selected here are qualitatively similar to the interactions between any four subtypes of high enough baseline abundance, the four subtypes utilized in this model have literature suggesting that they are each individually of clinical relevance. Two of bacteria involved in this model were selected for previously described potentiation of autism (Clostridia and Desulfovibrio) and the other two for the interactions with these two as described in the developing literature [3, 19, 21]

**Fig 1:**
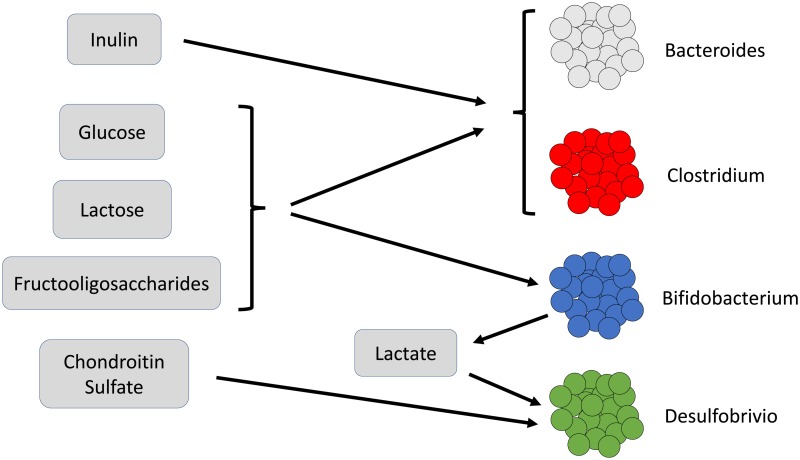
The metabolite relation between the bacteria.

There are two well-understood ways in which specific gut bacteria can cause problems for their host; the first is through permitting overgrowth of pathogenic bacteria and the second occurs when certain bacteria find their way out of the gut and into the bloodstream, causing bacteremia and sepsis. As a consequence, the majority of literature regarding specific microbes and the gut microbiome are centered on these two issues, neither of which is informative to the way the microbes interact within the gut in any other setting. Most of the bacteria in the gut are commensal and often provide benefits to their host by means of processing food into useful metabolites or breaking down harmful metabolites. Therefore, this model employs bacteria interactions at the level of metabolites that are generated, used, and competed for between the bacterial subtypes selected here, summarized in Fig 1 [22–27]. It is the paucity of literature that motivates the work presented here. Where biological experimentation has a limited ability to predict the interactions of individual microbes within the gut, a framework for simulation of bacterial communities based upon the limited knowledge of rules governing their interactions may provide valuable insight.

### 3.2 Model implementation

Our model simulates a competition between species of bacteria for resources in a constant fluid flow. Direct interactions between the bacteria have not been included at this time, but can be easily appended in future revisions. We begin by meshing the surface of the human ileum into 1x100 rectangular elements. The boundary conditions are periodic in the vertical direction and fixed flux at the horizontal entrance. Any agent that moves past the last horizontal element is removed from the simulation. Each element is an independent area of bacteria and metabolite concentrations. Bacteria can freely flow between elements as independent agents while metabolites are environmental variables. It is possible to model both metabolites and bacteria as agents, but such an implementation would increase the computation time significantly.

The spatial characteristics of the simulation are controlled by the velocity of the flow field, specified as a displacement per time step, and the probability of a bacterial colony becoming stuck in the mucosal membrane. The bacteria are translated down the simulation by the displacement each timestep. As the displacement is commonly less than the length of one element, bacteria may stay on the same element or translate to the next one. The probability for become embedded in the mucosal membrane (stuck chance) is defined as:

$$\begin{array}{c} \gamma =\gamma_{max}\left[\frac{1-N}{\left(N_{mid}+N\right)}\right] \end{array}$$ (1)

Where *γ*_*max*_ is the maximum probability, *N* is the number of bacteria locally, and *N*_*mid*_ is the median concentration provided. The stuck chance mechanism was added in the model since a constant, unidirectional flow would lead to washout of the bacteria. It has been inferred that bacterial populations resist washout through mixing in the fluid flow [28]. These additional effects can be added in future work. There is additionally a probability for bacteria to leave the mucus layer that is currently set at 10%. Bacteria already in the mucus layer also have a chance to become permanently embedded, a factor that is currently set to 5%. This effect prevents the complete washout of the bacteria without leading to unnatural bacterial accumulation.

Metabolite concentration is a function of position and the number of local bacteria. Metabolites are introduced each timestep at the entrance of the simulation. The concentrations were chosen based on the ability to support a stable and accurate baseline population of each of the four genera of bacteria modelled. The probability that bacteria consume a metabolite is based on the concentration of metabolites and of other hungry bacteria on the element. This probability is defined as a sequence of logic steps but can be simplified as a two parameter fitting between the concentrations with a random selection process. Hungry bacteria are defined as bacteria with an energy level below a threshold of 80 out of 100. Bacteria increase their energy by consuming appropriate metabolites. Each bacterial colony loses a constant amount of energy for each timestep. This loss is currently set so a colony with full energy can survive for 1440 timesteps without consuming metabolites.

Bacteria have a chance to reproduce based on their characteristic doubling time. For timestep that is a multiple of its doubling time, a bacterium can reproduce if it has more than 50 energy and is not space constrained. One of the resulting daughter cells has half of the initial bacteria’s energy and is released from the mucosal layer if the parent was embedded. The other cell retains the leftover energy and remains embedded.

Major assumptions made in GutLogo are summarized as:

- Unidirectional, constant velocity flow profile
- Negligible bacterial motility and turbulence
- Constant doubling-times throughout the simulation
- Same effective double times for all genera
- Bacteria are exclusively dependent on the nutrients simulated in the model
- Bacteria do not interact with each other directly, restricting population dynamics to competition for nutrients

These assumptions each represent initial simplifications that can be relaxed in future models. As is, our setup can still provide qualitative insights on various systems as demonstrated by proof-of-concept simulations.

Compared to the average lateral flow rate of 2.22 cm/min through the human gut, bacteria only move at approximately 0.12 cm/min by their own propulsion [29]. At about 5% of the lateral flow rate, the self-motility of bacteria can be neglected to simplify the computation. The fluid flow has been simplified into a constant unidirectional flow. Future models can improve on this model aspect by implementing a peristaltic flow.

Doubling-times may be variable due to a variety of factors, such as nutrient availability, but were kept constant in this model as a first-pass simplification. Future implementations of this model can easily incorporate a form of variable doubling-time.

One limitation of GutLogo is the assumption that the microbes exclusively rely on the metabolites in the simulation. When these nutrients are depleted, the cells in our model will die. In reality, there may be a plethora of alternative metabolites that the bacteria can consume, including mucin secreted by epithelial cells [30]. Available metabolites in the model could represent a variety of alternative metabolites that possess the same metabolic relationship with the microbes. Considering the complexity and variety of bacteria metabolites, we argue that this simplification is supported to maintain the desired model usability.

Microbial community dynamics can be quite sophisticated. We simplify the work of Weston et al. [19] by excluding direct interactions between bacteria. By doing so, we shift the focus towards competition for nutrients and increase the ease of adding additional microbes.

We also assume:

- There is some exchange between bacteria in the mucosa and bacteria in the lumen
- Each computational agent represents the same amount of bacteria (qualitative)

These assumptions act more as model parameters. We assume that there is some exchange between the mucosa bacteria and the bacteria that flow through the lumen. The extent of this exchange can be modified through modeling parameters. We also assume that some bacteria will colonize the mucous or crypts so extensively that exchange is negligible. We refer to these bacteria colonies as seeds. The chance of this happening is also a model parameter. Finally, we assume that each computational agent represents an equal number of bacteria. This allows for a more straightforward interpretation of the data, although by changing the ratio of the various agent breeds this assumption can be relaxed.

## 4 Methodology

### 4.1 Stability tests

**Fig 2:**
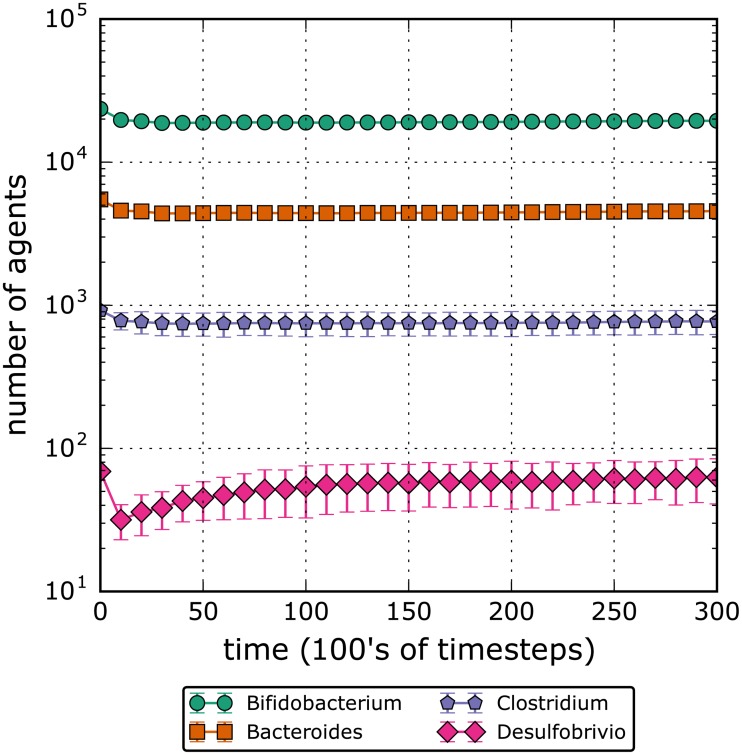
This graph shows the populations of the four genera of bacteria modeled over time. The control conditions are used for the initial conditions. One timestep is equivalent to 26.3 seconds, and a point is plotted every 1000 timesteps. The error bars represent the 95% confidence interval. We estimate that steady-state is reached at roughly the 10000 timesteps, which is approximately 3 days.

At model setup, the simulation was seeded with a normal gut population estimation based on Zhernakova et al.’s data on population-based metagenomics [31]. Bacterial colonies in the gut are modelled with relative population percentages of 78.43% *Bifidobacterium*, 18.27% *Bacteroides*, 3.06% *Clostridium*, and 0.23% *Desulfovibrio*. While the flow rate was set to biologically relevant values (as discussed previously), other model parameters, such as the bacteria doubling rate, were set to values such that the system would maintain an equilibrium state while not largely deviating from the initial conditions. By testing 100 iterations of the model on these settings, we found that 10000 timesteps was a sufficiently long time to reach an approximated steady-state, as shown in Fig 2.

### 4.2 Perturbation tests

Disease states in the gut may be induced by external factors that disturb the complex interplay of bacteria and metabolites, altering the steady-state population levels and leading to an immune response or an accumulation of toxins. To simulate how perturbations effect microbial dynamics, we determined three possible causes of deviation from a “normal” state of the gut: flow rate of material through the gut, change in diet, and probiotic consumption. For each perturbation, we ran 100 simulations. The initial conditions for each simulation are equivalent to the conditions in the control runs. We heuristically determined that 10000 timesteps was sufficient for the system to reach a steady-state. Because each of these timesteps equates to 26.3 seconds in our model, this time-frame is about three simulated days. Once the simulation runs for three days of simulation time, we introduce a perturbation for an additional simulated set of three days. We will refer to this time period as the perturbation interval. The perturbation varies depending on the type of perturbation we want to model. We then remove the perturbation and allow the system another simulated three days to re-stabilize.

*Bifidobacterium* have long been used as the active microbial ingredient in probiotics and are one of the most commonly administered probiotic agents [32]. To represent the addition of a probiotic, either 5000 or 10000 colonies of *Bifidobacterium* are added to the model during the perturbation interval every ∼3.5 simulated hours. Several species from the genus *Bifidobacterium* are commonly used probiotics and were incorporated in our previous model [19]. 5000 *Bifidobacterium* colonies are used to represent a low dose of probiotic, which we refer to as the lesser probiotic, and 10000 to represent a greater dose of probiotic. The *Bifidobacterium* cells are added to the model by setting the amount of *Bifidobacterium* at the inlet to either 5000 or 10000 units for one simulated timestep and allowing them to flow down the simulation.

To represent a change in diet we either doubled or halved the inflow of glucose during the perturbation interval. Initially, we set glucose inflow to 30 units per timestep to stay consistent with all control runs. We set the glucose inflow to 60 and 15 for the increased and decreased sugar diets, respectively.

Changing the flow rate can be used to model constipation or diarrhea. The fluid velocity can be altered by changing the displacement per timestep. In the healthy control this displacement is set to 0.278, and during the constipation perturbation it is decreased by a factor of three. Likewise, during the diarrhea perturbation, this value is increased by a factor of three. The 0.278 value was reached by factoring in the healthy residence time of food in the gut, the mean internal circumference of the intestine, and the ratio of the intestinal length to map length in our model.

## 5 Results

### 5.1 Population response to probiotics

To represent the intake of probiotics, which are commonly sought for chronic gut-related maladies, we introduced a probiotic of bifidobacteria flowing into the gut per tick. During the three day period in which probiotics are consumed, the population of *Bifidobacterium* increases as expected, while the populations of *Clostridium* and *Bacteroides* decrease. These populations decline due to the increased competition for the nutrients glucose, inulin, lactose and fructooligosaccharide, all of which are also consumed by bifidobacteria. Although *Bifidobacterium* cells produce lactate, which is an energy source for *Desulfovibrio* species, our model suggests that probiotics have no statistically significant effect on the *Desulfovibrio* population. This is most likely for two reasons: 1) The population is already dominated by bifidobacteria and all available carbohydrates are already being consumed prior to the addition of the probiotic. Thus, adding bifidobacteria only marginally increases the amount of carbohydrates that are metabolized by bifidobacteria as *Bifidobacterium* cells must compete amongst themselves for carbohydrates. Therefore a large increase in bifidobacteria only marginally increases lactate production. And, 2) the parameter for lactate production is set too low to make a significant impact in consideration of the previous argument. Thus, dosing of probiotics creates only small fluctuations in the lactate production, rather than a significant net increase in lactate as expected. After treatment of probiotics has concluded, all populations returned close to their initial population levels, suggesting that the effects of probiotics (if the species being administered is already present in the gut) are transient.

**Fig 3:**
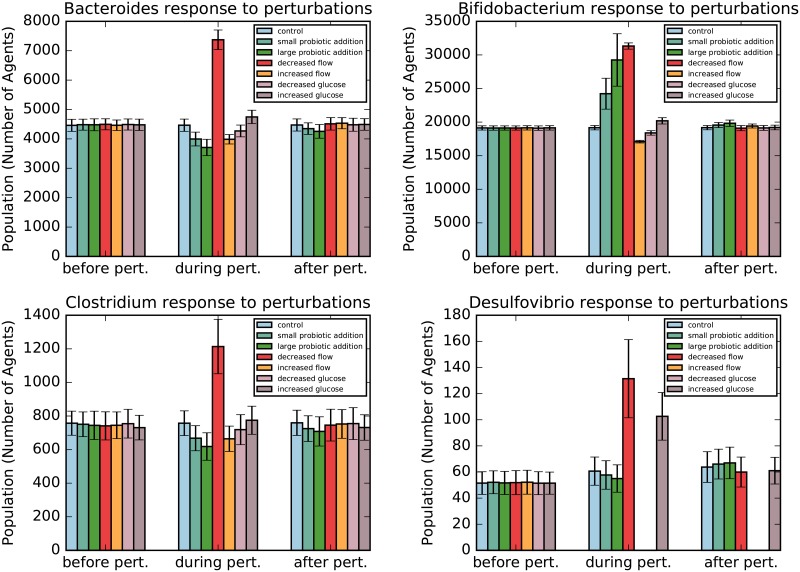
Steady state population levels of *Bacteroides*, *Bifidobacterium*, *Clostridium* and *Desulfovibrio* before, during and after each individual system perturbation. The error bars represent a 95% confidence interval.

**Fig 4:**
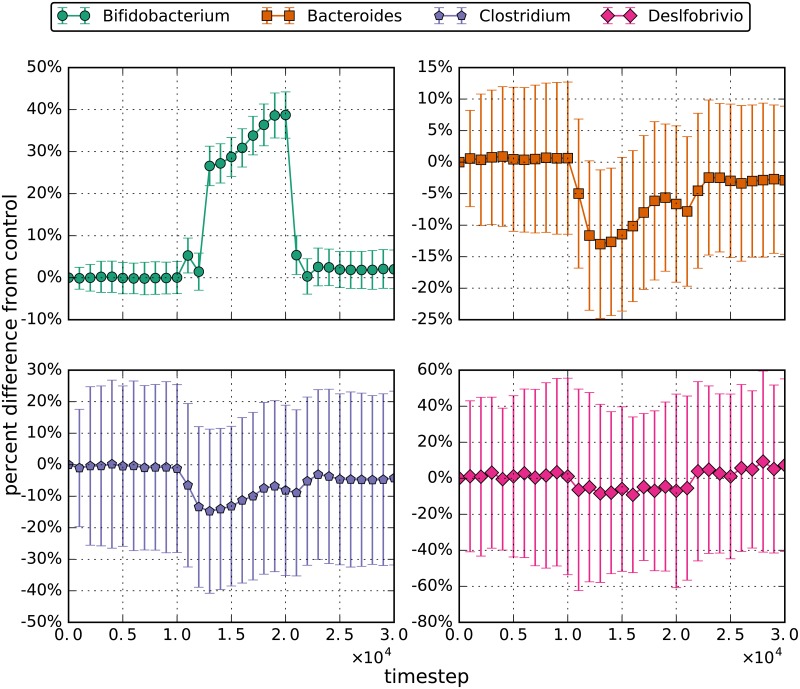
Percent difference from the control for the the lesser probiotic (low dose) simulations. The errorbars are the 95% confidence interval for the distributions. During the perturbation interval of the simulation, 5000 bifidobacteria enter the left side of the simulated area every 3.5 simulated hours or 480 timesteps.

The abundance of bifidobacteria and clostridia in the gut tend to be inversely correlated [33]. Furthermore, it has been demonstrated that the growth of *Clostridium difficile* was significantly inhibited when co-cultured with *Bifidobacterium longum* JDM301 *in vitro* [34]. In congruence with these findings, results of our model suggest that the *Clostridium* population will decline with a *Bifidobacterium* probiotic (see Figs 3 & 4). This suggests that the negative impact that bifidobacteria has on *Clostridium* populations is likely, in part, due to competition for nutrients. In a separate study, certain species of *Bifidobacterium* were shown to outcompete *Bacteroides thetaiotaomicron* for food in co-cultures [35]. This would likely translate to reduced populations of *Bacteroides* in the human gut after *Bifidobacterium* probiotics, a result that was also captured in the Gutlogo model. While our model suggests only transient effects from probiotics, there are some scenarios in which a permanent shift in the population steady state is conceivable. For example, for a more complicated microbial community, there could be multiple possible steady states. In such a scenario, a population shift due to a perturbation may drive the community to settle into a different steady state upon the removal of the perturbation. Alternatively, it is possible that the influx of one bacteria via probiotics could result in the die-off of another species due to nutrient competition, microbial interaction dynamics or a host immune response. Furthermore, the introduction of a new bacterial species via probiotics could result in a colonization event, precipitating a long term change in the microbiota.

### 5.2 Population response to different flow rates

**Fig 5:**
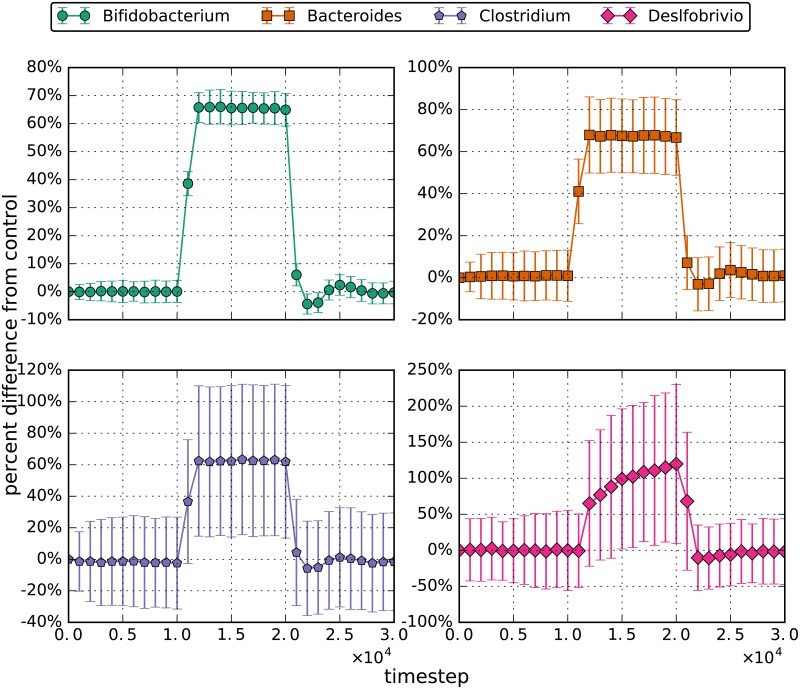
Percent difference from the control for the decreased flow tests. The errorbars are the 95% confidence interval for the distributions. Once the flow is decreased to 1/3 of its initial value, the resultant bacterial populations all dramatically increased, although *Desulfovibrio* increased by a larger margin than the other genera. When conditions were reverted to initial values, the populations of the bacteria fell to their initial steady states.

To test the effects of flow rate on the gut microbiota, we first decreased the rate by a factor of three to simulate the intestine during constipation. In this scenario, the population levels of all bacteria increased substantially (see Figs 3 & 5), largely due to an increase in residence time of the bacteria. When the flow rate is returned to normal, we see that the population levels readjust to roughly match the control values. This result aligns with the findings of Zoppi et al. who found that constipation was accompanied by increases in the populations of *Bacteroides*, *Clostridium*, and *Bifidobacterium*, although the increases were not as substantial as those seen in our simulation [36]. It is possible that a host immune response would limit the population increase to more moderate levels, explaining our model’s overestimation of the shift. A combination of immune response and sustained increase in bacteria levels would have the potential to induce far-reaching health effects.

The text on decreased flow rate was complemented by a simulation of diarrhea, in which the flow rate was tripled for a period of three days. In this case, the populations of *Bifidobacterium*, *Clostridium* and *Bacteroides* decrease marginally and return to control values when the perturbation has ended (see Fig 3). These results conform to clinical findings that patients with diarrhea have lower populations of *Clostridium* and *Bacteroides* compared to healthy controls [37]. On the other hand, the *Desulfovibrio* population completely dies off during this constipation period since lactate, which is naturally at low levels within the simulation, is flushed out to levels that cannot sustain the population. While it is possible this die-off would occur in the real system, it is more probable that the *Desulfovibrio* population would be able to persist at low levels by shifting its metabolism to require less nutrients or utilizing alternative metabolites. Improvements to this model could be made to account for bacteria functioning in lower nutrient conditions. A simple improvement would be to add additional nutrient sources, however this does not address the root of the problem. A better approach might be to remove the rule that limits carbohydrate consumption to discrete units of one, and replacing the consumption rate with a function that takes the external nutrient concentration as a variable. Such a function could saturate when nutrients are plentiful, but would still provide some flux of carbohydrates at low nutrient conditions. Energy provided to the cell would have to be proportional to this flux. Additionally, the amount of energy consumed each time step and the rate of reproduction could also be decreased if nutrient flux is reduced, thus improving the life-span of the cell in low nutrient conditions. We leave such improvements to be implemented in future models.

### 5.3 Population response to altered glucose levels

One important factor to consider when investigating microbiome community dynamics is the influence of the diet. Turnbaugh et al. demonstrated that bacterial population levels shift within one day in the guts of humanized gnotobiotic mice when switching from a low-fat plant-based diet to a diet high in fat and sugar [38]. In the GutLogo simulation, the high-glucose diet saw a marginal increase in *Bifidobacterium*, an insignificant change for *Clostridium* and *Bacteriodes* populations and a sharp rise in the *Desulfovibrio* population, despite the fact that *Desulfovibrio* does not utilize glucose in this model. Similar to the findings of Turnbaugh et al., these shifts occurred rapidly. Importantly, this result also suggests that prebiotics may have effects beyond the targeted bacterial species due to metabolite production and ecosystem dynamics, and that dietary changes could dramatically alter the gut microbiota in unexpected ways. A more thorough investigation of community dynamics is necessary to fully understand the potential effects of prebiotics on the microbiome. Similar to the increased-flow simulation, the low-glucose diet resulted in a complete die-off of *Desulfovibrio* cells. The elimination of an entire genus is an unlikely outcome from such a minor dietary change, so it is again probable that *Desulfovibrio* cells would subsist on lower nutrient levels or alternate fuel supplies and persist at a nonzero population level. Such questions can be addressed in future models, after making improvements as discussed previously.

**Fig 6:**
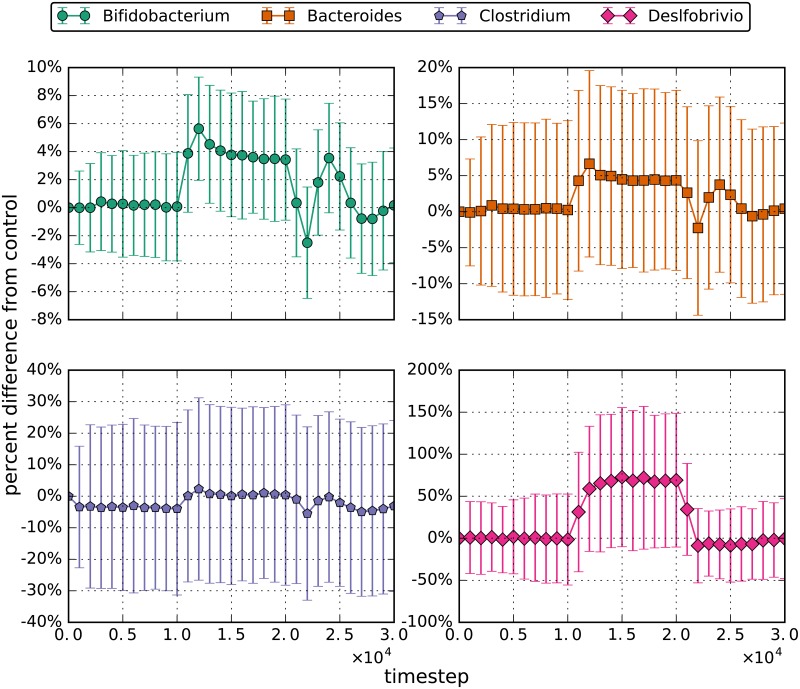
Percent difference from the control for increased glucose simulations. The errorbars are the 95% confidence interval for the distributions. The amount of glucose entering the gut every tick started at 30 and doubled to 60 during the perturbation phase.

As an extension of this increased-glucose experiment, each bacterial population was tracked over the simulation timeframe and normalized to a constant-glucose control. These results are shown in Fig 6, with timestep 10000 marking the instance where glucose inflow increased from 30 to 60 units per timestep. The results show a slight increase in populations of *Bifidobacterium*, *Bacteroides*, and *Clostridium* while the glucose concentrations were increased. Once the glucose concentration was returned to the original levels, the results also show a temporary decrease below steady-state populations before returning to steady-state populations for the same genera. *Desulfobrivio* populations increase on average by 70% with increased glucose. This is due to increased lactate concentrations produced by the *Bifidobacterium*. These effects are similar to those seem in the decreased flow rate simulations and suggests that short changes in glucose intake will not affect gut microbial populations.

### 5.4 Spatial considerations

**Fig 7:**
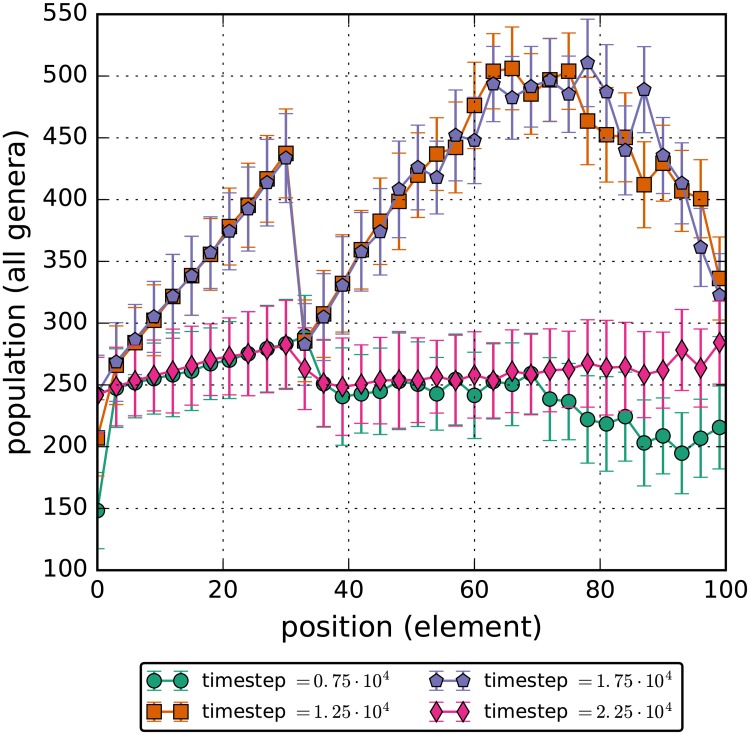
Total number of bacteria in each of the elements for the decreased flow rate simulations. The flow rate for carbs and bacteria is decreased to 1/3 of the unperturbed rate for the timesteps between 1.0 ⋅ 10^4^ and 2.0 ⋅ 10^4^. Errorbars contain the 95% confidence interval for the simulations. During this time, bacteria cluster in higher numbers in the 40th to the 80th element.

**Fig 8:**
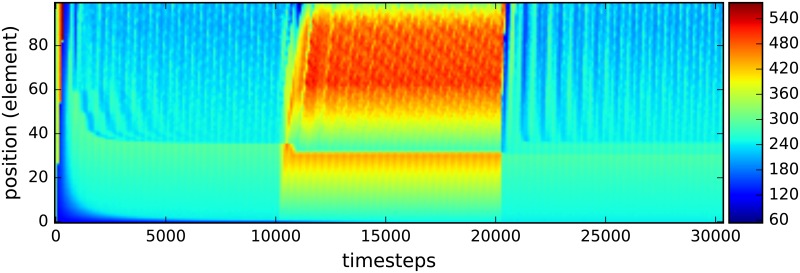
Heatmap of the total number of bacteria in each of the elements for the decreased flow rate simulations. The flow rate for carbs and bacteria is decreased to 1/3 of the unperturbed rate for the timesteps between 1.0 ⋅ 10^4^ and 2.0 ⋅ 10^4^.

An additional benefit of this simulation architecture is the capability to see shifts in the spatial distribution of bacteria under specific external forces. As a demonstration of this feature, the total bacteria population was plotted as a function of the discrete spatial region at four key timesteps. Once before the perturbation, once right after the perturbation started, once right before the perturbation ended and once after the perturbation ended. (Fig 7). Of primary interest in this plot is the spatial distribution of the two intermediate timesteps. Not only does the total number of bacteria increase when the flow rate through the gut is decreased; the bacteria also aggregate heavily within regions 50 to 90 of the model and in the foremost region of the map. Accumulation in the early rows is most likely due to the abundance of nutrients flowing in through those patches, however there are no inherent differences between row 40 to 80 compared to the other patches. This suggests that the bacteria inherently optimize to occupy those positions, highlighting the need to investigate both shifts in bacteria composition and changes in spatial distribution in relation to disease. Additionally, a population heatmap as a function of position and timestep is included. (Fig 8). It verifies the results discussed above.

**Fig 9:**
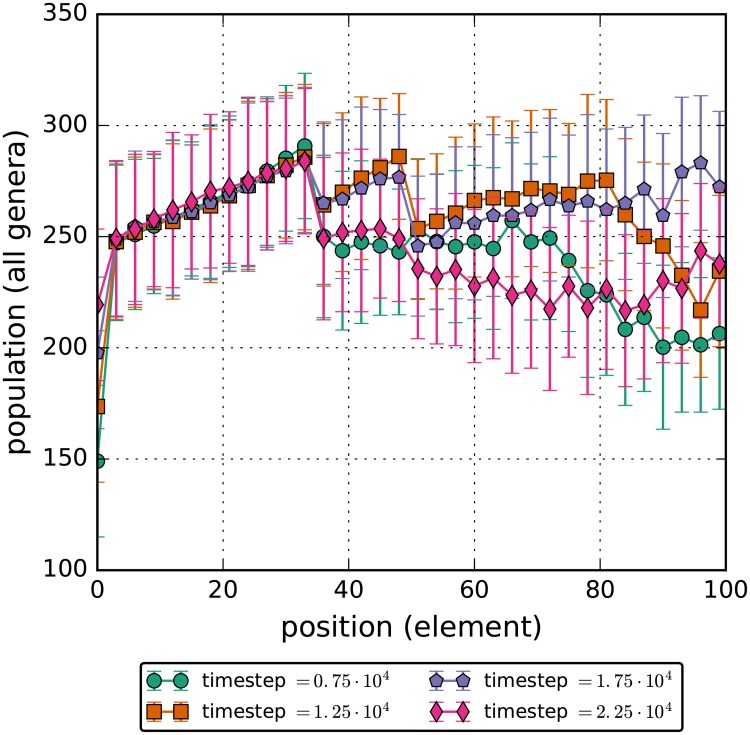
Total number of bacteria in each of the elements. The glucose flux is increased to double of the unperturbed rate for the timesteps between 1.0 ⋅ 10^4^ and 2.0 ⋅ 10^4^. The increase in glucose results in an approximately equal increase in bacteria populations throughout the simulation length. The populations rapidly return to pre-perturbation levels after the glucose levels are returned to initial conditions.

**Fig 10:**
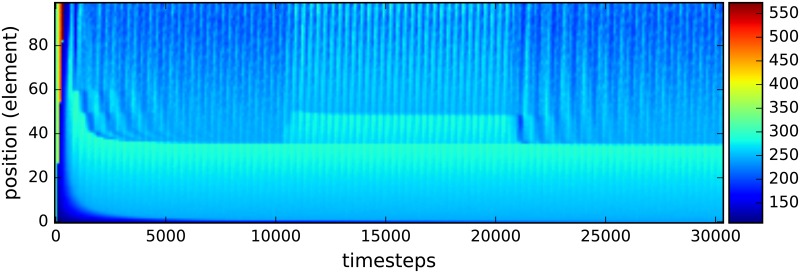
Heatmap for the total number of bacteria in each of the elements. The glucose flux is increased to double of the unperturbed rate for the timesteps between 1.0 ⋅ 10^4^ and 2.0 ⋅ 10^4^.

These drastic changes can be easily compared to the spatial and heatmap plots, (Figs (9) and (10)), for the perturbation of increased glucose which has the total population by discrete spatial region barely change in a statistically significant way even during the perturbation. The only meaningful change during the perturbation is the increase of population in the final quarter in the model. This is most likely due to the fact that with more glucose entering the ileum, more of it can reach the end which allows the bacteria to eat and so die less frequently.

Due to the emergent complexities of a multi-agent system, GutLogo and similar agent-based simulations have a unique ability to represent the stochastic nature of the microbiome while also maintaining a large degree of customizability and user accessibility. This allows for the elucidation of more insightful and clinically relevant behavior, even with a simple 4-bacteria model. These initial tests help to validate GutLogo as a useful modeling framework and provide a brief look at its utility in studying the complex behavior of bacterial systems.

## 6 Conclusions

Agent-based models are well-suited for representing human physiology, avoiding the computational complexity of partial differential equations while presenting a relatively accurate approximation of how actual biological systems behave. GutLogo, a NetLogo-based simulation of the human gut microbiota, incorporates four bacterial species and six metabolites to present a simplified representation of bacterial interactions on the human intestinal lining. By incorporating fluid flow and spatial differentiation, this program builds on previous work and allows for a more accurate study of the relationship between gut perturbations and human disease. As a modeling framework, GutLogo can be modified by independent parties to simulate any number of bacterial systems or external perturbations. These initial tests help to establish a validated, expandable platform that allows researchers to virtually investigate complex interactions between the microbiota and the human host that would be difficult or unethical to study in a clinical setting. The program also demonstrates the evolution of bacterial distributions along the length of the gut as a function of time, adding another level of sophistication that can be used to observe previously unseen phenomenon and suggest promising areas for further investigation.

## Supporting information

S1 Data Raw data. Raw data from the simulations.
(ZIP)

S2 Data Formatted data. The averages and other extracted values from the raw data.
(ZIP)
